# Sustainable Development versus Extractivist Deforestation in Tropical, Subtropical, and Boreal Forest Ecosystems: Repercussions and Controversies about the Mother Tree and the Mycorrhizal Network Hypothesis

**DOI:** 10.3390/plants13091231

**Published:** 2024-04-29

**Authors:** Tomas Gabriel Bas, Mario Luis Sáez, Nicolas Sáez

**Affiliations:** 1 Escuela de Ciencias Empresariales, Universidad Católica del Norte, Coquimbo 1780000, Chile; nicolas.saez01@alumnos.ucn.cl; 2 Facultad de Humanidades, La Serena University, Coquimbo 1700000, Chile; mario.saez@userena.cl

**Keywords:** native forests, extractivist deforestation, biodiversity, ecosystem, coopetition, cascade mother tree, mycorrhizal, public policies

## Abstract

This research reviews the phenomenon of extractive deforestation as a possible trigger for cascade reactions that could affect part of the forest ecosystem and its biodiversity (surface, aerial, and underground) in tropical, subtropical, and boreal forests. The controversy and disparities in criteria generated in the international scientific community around the hypothesis of a possible link between “mother trees” and mycorrhizal networks in coopetition for nutrients, nitrogen, and carbon are analyzed. The objective is to promote awareness to generate more scientific knowledge about the eventual impacts of forest extraction. Public policies are emphasized as crucial mediators for balanced sustainable development. Currently, the effects of extractive deforestation on forest ecosystems are poorly understood, which requires caution and forest protection. Continued research to increase our knowledge in molecular biology is advocated to understand the adaptation of biological organisms to the new conditions of the ecosystem both in the face of extractive deforestation and reforestation. The environmental impacts of extractive deforestation, such as the loss of biodiversity, soil degradation, altered water cycles, and the contribution of climate change, remain largely unknown. Long-term and high-quality research is essential to ensure forest sustainability and the preservation of biodiversity for future generations.

## 1. Introduction

Tropical, subtropical, and boreal forests are critical components of the different ecosystems of the Earth, supporting an astonishing variety of species and facilitating the transition to an essential ecological balance [1,2]. Forests sequester approximately 30% of Earth’s carbon [3]. It is often considered a net carbon sink to neutralize atmospheric CO_2_ along with phytoplankton in the oceans [4]. However, tropical, subtropical, and boreal forests face numerous problems, mainly related to the extractive culture generated in many cases by mining, oil, real estate, and forestry industry consortiums that usually cause massive and often indiscriminate deforestation [5]. Natural resources are an essential pillar for the development and survival of humanity, as they provide the raw materials and energy necessary for its development, so the key is to find a balance between the exploitation of natural resources and socio-economic growth accompanied by sustainable development [6]. The instability caused by deforestation could have a global impact on the weakening of forest biodiversity and the surrounding environment that comprises aerial, surface, and underground ecosystems [7]. Below the ground, there is a geological stratum that extends from the surface of the earth to the water counter of the first free aquifer, known as the ‘vadose zone’, and has key functions within the biosphere [8]. It would serve as a hydrological reservoir, facilitating the supply of water to plants and the atmosphere, simplifying the exchange of nutrients, and facilitating the survival of the ecosystem [9,10].

Suzanne Simard’s research explores a hypothesis that attempts to explain the possible negative effects of intensive deforestation by extracting what they call “mother trees”, referring to groups of older trees that play a protective role over the rest of the trees in their environment [11]. According to her hypothesis, the roots of these trees would be able to collaborate in the exchange of nutrients by forming an intricate communicational network with mycorrhizal fungi sharing some nutrients, particularly carbon and nitrogen [12,13,14,15,16,17,18,19]. Therefore, according to Simard, the roots of older trees would constitute a network with the fungal community located on their roots that would allow plants to exchange some essential nutrients for their survival and growth, and, in turn, the fungi would help the host plants maximize the transfer of some fundamental nutrients [11,17,18,20,21,22,23,24,25]. Their research suggests that the removal of these “mother trees” could break this protective balance between roots and mycorrhizal fungi, affecting the rest of the forest ecosystem, particularly the younger trees. However, the results of this research are not unanimous in the international scientific community and opinions are divided. Some researchers do not adhere to this hypothesis, arguing that such a dependence between fungi and tree roots as suggested by Simard [26] has not been fully demonstrated. Other research argues that mycorrhizal fungi are not extensions of roots but rather organisms that might establish different strategies than host trees. There may even be eco-physiological differences between the different types of mycorrhizae, which may indicate strategies and functions that are unique to them [27].

It is important to note that mycorrhizal fungi are divided into two categories: ectomycorrhizae and endomycorrhizae, which include arbuscular mycorrhizae. These fungi establish symbiotic relationships with more than 80% of terrestrial plant roots, providing them with nutrients synthesized from the soil with which they form interaction networks between two or more plants linked by a fungal symbiont, which are called common mycorrhizal networks or even the “wood-wide-web” [28,29,30,31,32]. These networks may even facilitate interactions between plants, including the transmission of aphid-induced diseases and signals and the activation of chemical defenses in neighboring plants [33]. More recently, molecular research on the identification and gene functions of arbuscular mycorrhizal fungi is being carried out, which will improve our understanding of soil/plant interaction mechanisms in different forests [33,34,35].

In this amalgam of interconnections, competencies, and interrelationships of the forest ecosystem, we propose the notion of ‘coopetition’, originating from the world of management but adaptable to the ‘web of forests’. Coopetition as a theoretical concept originates from inter-institutional cooperation, both bilateral and multilateral [36,37]. In this dynamic, the same authors define coopetition as simultaneous cooperation and competition between two or more competitors. Coopetition belongs to the highest-cost interorganizational relationships, and this results from the contradiction of logic that coopetition is based on trust and conflict at the same time [38]. If we transfer this to nature, the concept of coopetition applied to the forest refers to the fact that trees are capable of competing and cooperating at the same time within an ecosystem for certain resources. The ecosystem could be defined as any element or variable that is associated with another in a system and dependent, and consequently, the relationship between the components preserves the overall survival of the ecosystem [39,40,41]. Based on this logic, it can be said that, in general, an ecosystem is dynamic, which means that the ecosystem constantly incorporates new elements or variables with continuous feedback, allowing for increasing the predominant biodiversity in a given space [42].

Another concept directly related to interrelationships in forests is resilience and its different variants. This research refers to how anthropogenic changes can affect forest disturbance patterns, potentially reducing their capacity to provide ecosystem services [43,44]. This alteration can also influence the distribution and adaptation of forest-dependent species due to abrupt habitat changes [45]. Combined disturbances can change forest trajectories, and an increased frequency of disturbances can weaken forest resilience [46,47]. In close relation to resilience, some studies estimate that tropical forest ecosystems, for example, are capable of actively configuring their environmental parameters, through feedback mechanisms that operate on various spatial scales [48,49]. The same author values these mechanisms as crucial to understanding what is known as hysteresis, which is a form of stability forged from dependence on a previous historical state; hence, its proximity to resilience and a certain capacity that some ecosystems could have to resist change despite the loss of their trees due to extractive deforestation, managing in some cases to recover and likely even adapt to potential climate changes [47]. It is interesting to see how, through resilience, some forests can adapt to disturbances, whether anthropogenic or even natural and in many cases conserve their essential structures and functions [16]. However, it is important to note that some studies show that while tropical forests could recover in the medium term from potential extractive deforestation, in the long term, they would also lose their resilience [48].

It is imperative to note that healthy forests contribute to climate regulation, carbon sequestration, and the water cycle, among other aspects of the ecosystem, making them vital to the well-being of the planet [2,18]. As the human influence on the Earth expands, native forests suffer a highly significant impact, which is reflected in their gradual decline [50,51,52]. Preserving and restoring the integrity of different types of native forests is essential to strategically address the biodiversity crisis, try to curb climate change, and promote medium and long-term sustainability [53]. The preservation of forest integrity must be fundamental to global and local environmental strategies, complementing current efforts against deforestation and, in contrast, in favor of reforestation [54]. Conservation efforts should consider the complex relationships within forests themselves, emphasizing the importance of ecosystems and their impact when forests are completely cleared or when fires (intentional or otherwise) destroy life in their path [55,56].

In many developing countries, public policies aimed at protecting natural resources are generally poor, the institutional framework does not function adequately, and governance is often dominated by high levels of corruption [57,58]. In such an environment, it is very complex to generate regulations that limit the overexploitation of forests generated by the extractive practices of large companies, many of them foreign, which take advantage of the laxity of institutions and governance, circumventing existing regulations. This generates unprecedented overexploitation of native forests (tropical, subtropical, and boreal) and the loss of tree cover and, therefore, the death in some cases of all the biodiversity generated by woodland [59,60,61]. This phenomenon of overexploitation is defined as extractivism, and its conceptual framework covers a wide spectrum of research domains related to the impact on natural capital [62,63,64]. This practice encompasses an intricate interaction of activities framed by power asymmetries and, in many cases, institutional weakness. Corruption is a strong element and often plays a leading role in decisions made concerning the exploitation of natural resources with socio-economic and socioecological impacts. In many of these cases, the beneficiaries tend to be few, to the detriment of populations with fewer resources that inhabit the exploited areas [65,66]. The increase in the monetary values of raw materials during the transition of the last millennium generated an increase in the extractivist phenomenon in Latin America (rich in minerals, oil, aquatic resources, forests, and agriculture), fundamentally affecting the tropical and subtropical woodland of this geographic forest region [49,67]. This voracity occurred regardless of the economic ideology adopted by the respective national administrations, be it ‘neoliberal’, ‘post-neoliberal’, or ‘neosocialist’ [68]. In this context, the term extractivism is elucidated as the extraction and excessive and abusive use of any natural resource, regardless of the strategies and actions used [69]. Therefore, it is essential to implement public policies that limit this extreme practice and prevent it from being a generic term used exclusively for mining, gas, and oil [70]. This would prevent the looting and irreparable damage caused by extractivism to native tropical, subtropical, and boreal forests, which simultaneously affects all the biological organisms and the minerals that depend on them.

In light of the documentary exploration analyzed, the following research questions arise.

- Is it possible to address the controversies surrounding the ‘mother tree’ hypothesis, along with the lack of conclusive research results from its detractors, and reach a consensus on sustainable forest development?
- Could genetic engineering techniques provide a deeper understanding of the intricate relationships between ‘mother trees’, mycorrhizal fungi, and younger trees within forest ecosystems?
- How can we address the controversies surrounding cascading phenomena and the resilience of forest ecosystems while evaluating the effectiveness of public policies to mitigate extractive deforestation and promote sustainable forest management practices?

In this context, two objectives are visualized.

- Stimulate greater commitment within the international scientific community to investigate the potential impacts of extractive deforestation based on the lack of knowledge of many of the reactions triggered by altering the ecosystem and its biodiversity.
- Examine the phenomena of coopetition, cascade, and resilience within forest ecosystems, to encourage the generation of public policies that mitigate the potential effects of extractive deforestation and promote sustainable forest management practices.

For the analysis of objectives, a methodology was implemented with a qualitative bibliographic documentary approach [71]. This approach involves a single search phase. The main effort involves a systematic literature review, conducted with meticulous care to compile a comprehensive collection of research that can effectively address the research question and stated objective. The review analyzed Suzanne Simard’s major research topics, including the influence of mother trees on intercommunication, coopetition, the symbiotic function of mycorrhizal fungi, and the resulting impacts on natural capital and surrounding biodiversity. Similarly, a bibliographic search was conducted for the years preceding Simard’s study period to better understand the antecedents that led to the theory of mother trees and the interaction of mycorrhizae (ecto- and endomycorrhizae). Additionally, a bibliographic search was completed for mycorrhizal strains by forest geographic region, as well as the latest studies in molecular research, genetic engineering, and biotechnology that included them. The study examined deforestation, with a particular focus on extractive practices that cause significant forest degradation and loss of biodiversity. Finally, a third aspect concerns the analysis of the significance of public policies, which are often inadequate in developing countries due to poor institutional quality. These countries are home to boreal, tropical, and subtropical forests, and suffer from inefficient governance, often contaminated by corruption that allows corporations to exploit forests and other resources in an inefficient way.

To enhance the search’s effectiveness, we selected appropriate documentary sources, bibliographic databases, and search engines based on the field or subject area. These included Science Direct, Agricola, Compendex, Derwent, Statistics Canada, Scopus, Web of Science Core Collections, Google Scholar, Innovation Index, and GeoIndex. To expand the exploration of search systems, we selected several research tools based on Gusenbauer and Haddaway’s [72] widely used and interdisciplinary approach. The following academic databases were searched: AMiner, ACM, arXiv, BASE (Bielefeld Academic Search Engine), CiteSeerX, DBLP (Digital Bibliography & Library Project), DOAJ (Directory of Open Access Journals), IEEE Xplore Digital Library, JSTOR, Microsoft Academic, Semantic Scholar, SpringerLink, Wiley Online Library, WorldCat, and WorldWideScience.

Syntax analysis was performed to determine the logical structure of high-level concepts in a query. Concepts are automatically extracted and assigned to entities for entity expansion. Data post-processing operations, such as search and derivation filters, are then applied to these entities [73,74]. The selections were combined to avoid any potential data duplication. The literature search was conducted based on the selection criteria, utilizing Boolean relationships between keywords through logical operators such as AND and OR. The approach for the automatic formulation of queries, specific to Boolean queries, is similar to the processes used by information specialists, but without the use of specialized software. The search was structured methodically, taking into account the level of sensitivity (retrieval rate) and specificity (precision rate). The research approach was determined by defining the research question and objective, which allowed focused information retrieval [75]. The framework consists of five steps: query logic composition (1), entity extraction (2), entity expansion (3), keyword mapping (4), and post-processing (5) [73]. According to some authors, a concise representation of the required information, either as a single word or a short phrase (e.g., ‘exploitative extractivist practices’), is necessary for the literature search strategy [75]. To broaden the scope of the investigation, certain concepts were split. For example, the term ‘extractivist practices’ was split into ‘extractivist’ and ‘practices’ based on semantic cues such as Word/cloud and Word/frequency’ [76]. Each word or phrase within a concept or semantic key serves as a synonym or closely related term. Relevant connections are crossed as necessary to obtain totals and frequencies. Boolean operators and parentheses were used to achieve the desired result, along with precise expressions and appropriate field codes. The text has been edited to ensure grammatical correctness and adherence to the desired characteristics. 

A systematic literature review was conducted using Natural Language Processing (NLP) through ‘Vectara,’ a free application for data analysis. 

The exploration covered the time period from 2015 to 2023 and involved defining and searching for keywords in the aforementioned databases. The time period of 2015–2023 was chosen to cover recent research on the topic, with preceding years added to include earlier studies that contributed to the development of Suzanne Simard’s hypothesis. The frequency of defined keywords in the titles and abstracts of the raw selection was calculated, with a minimum of three occurrences per abstract required. The compound keywords used were as follows: “Mother trees”, also known as “Guardians of ecosystem resilience”, are a key component in the “Suzanne Simard hypothesis” of symbiotic relationships between fungi and plants. These relationships involve the exchange of carbohydrates and water and are crucial for the underground networks of biodiversity and mycorrhizae, including arbuscular mycorrhizae, endomycorrhizae, and ectomycorrhizae. Deforestation, extraction, and biodiversity loss have a significant impact on these networks. It is important to understand and protect these relationships for the health of our ecosystems. The following topics are covered in this text: Mycorrhizae and genetic engineering, biotechnology and mycorrhizae, molecular research on mycorrhizae, rhizome and nitrogen collection, coopetition in the forest, dynamic ecosystem and biodiversity, forest resilience, resilience and disturbance, healthy forests, and overexploitation and resource depletion. The text provided appears to be a list of keywords or phrases related to forest biological diversity, including different types of forests, natural capital, deforestation, and nutrient exchange. The language used is generally clear and objective, but there are a few instances where the meaning could be made more precise through the use of subject-specific vocabulary. The text is grammatically correct and follows a logical structure, but it is not clear what the purpose of the list is or how the different keywords are related to each other. Therefore, no changes have been made to the text, but it would be helpful to have more context or information on how this list will be used or what it is intended to convey. The text discusses various topics related to extractivism, including the alteration of cascading nutrient exchange, vadose zone and water sequences, the modification of water cycles, cascading ecological effects, the importance of public policies, the regulation of the extractivist framework, institutions and governance, extractivism versus sustainability, regulation of extractivist phenomena, and corruption. During the initial phase, a comprehensive repository of 1472 documents was compiled, including research articles from journals indexed in Clarivate and Scopus, reviews, books, book chapters, and conference proceedings. It is important to note that this sample of 1472 documents is representative of the information analyzed but does not encompass all existing documents in the databases consulted.

- A meticulous manual selection process was used to refine the collected resources, eliminating duplicates and irrelevant entries. Only one instance of identical documents found in different databases was retained. This process resulted in a final collection of 370 documents.
- Selection based on title and keywords: The retained documents were subjected to a new phase of scrutiny. Their titles and keywords were subjected to a meticulous evaluation guided by an inclusive question: “Does the research contribute to reflect on the close relationship between the forest ecosystem based on Suzanne Simard’s hypothesis, mother trees, mycorrhizae, coopetition, and the unsustainable impact of the extractivist phenomenon on forests, the inability of public policies, institutionalism, and governance to safeguard the fragile balance of biodiversity?”
- Evaluation through summaries: In this phase, the summaries of the remaining papers were reviewed against the same evaluative question as in the previous step.
- Reading the full text and determining relevance: the full texts of the selected papers were diligently obtained and thoroughly studied. The evaluation question of the third step was reviewed to assess the alignment with the research objectives.

- During the comprehensive content analysis of the full text, we identified two fundamental areas influenced by the central research thrusts: the positive impact of mother trees on the larger interconnected forest ecosystem and the negative consequences of extractivism caused by indiscriminate deforestation. Keywords included “Deforestation”; “Extractivist practice”; “Biodiversity”; “Resilience”; “Suzanne Simard”; “Mother trees”; “Mycorrhizal networks”; “Mycorrhizal molecular research”; “Sustainable development”; “Importance of public policies”; “Institutions”; “Governance”; “Regulation of extractivism phenomena”; and “Corruption”. The formulation of queries for systematic literature review searches can be automated without the need for computer specialists or specialized software, as demonstrated by the use of Vectara. This method can lead to efficient searches and provide an overview of the types of studies recovered [72,73].
- Table 1 shows the frequency of each keyword reflected in the different databases used, with a total of 1472 occurrences.
- A high frequency indicates that the topic is widely discussed or emphasized in the literature. This indicates that a particular topic is of interest, concern, or research within the field. For instance, if the phrase “Impact of deforestation, extraction, and biodiversity” appears frequently, it implies that these issues are central concerns in the analyzed documents.
- A moderate count suggests that the topic is relevant, but not as dominant within the literature. It may be more specialized or focused on specific aspects of the broader field.
- A low frequency indicates that the topic is not a major focus within the literature. A low count may indicate that the topic is niche, emerging, or is not extensively covered in the literature. It could also suggest areas where further research is needed or topics that are less prioritized within the current scope of discussions.

## 2. Theoretical Framework

This research seeks to reflect and raise awareness about the potential impact that extractive deforestation could have on biodiversity and the interaction of tropical, subtropical, and boreal forest ecosystems and about more sustainable development. The rationale revolves around the conceptual and empirical controversy on the hypothesis proposed by Suzanne Simard’s research on mother trees and their possible influence on both the forest environment and mycorrhizal networks. The analysis is not limited only to the hypothesis developed by Simard but also contains some research contributions that oppose this hypothesis, thus allowing research with different points of view. However, the contribution of this study is broader as a whole, strengthening socioeconomic needs and interests, complementing the actions and responsibility to generate public policies and institutions that support the preservation of the tree resource and the rich and unique natural capital in the biodiversity of each geographic forest region considered for future generations. This is fundamental due to the lack of conclusive data for both positions.

### 2.1. Tropical, Subtropical, and Boreal Forests and Importance for Biodiversity

Native tropical, subtropical, and boreal forests are all critical to the ecological balance of the planet as they have unique and specific characteristics in terms of biodiversity, ecology, and carbon sequestration [1,77,78,79,80,81]. These differences underscore the complexity of forest ecosystems based on some attributes such as diversity, abundance, transition, resistance, and adaptation [82,83,84,85,86].

Tropical forests are located in an area where they average high temperatures and abundant annual rainfall, soil with very poor nutrient availability, and very high levels of biodiversity and growing seasons that generally extend throughout the year [87]. The tropical forest ecosystem plays a vital role in regulating the global climate and is essential in the water and carbon cycle [88,89]. These forests are geographically close to the equator in regions such as the Amazon rainforest in South America and the Congo rainforest in Africa, which belong to some of the most biodiverse ecosystems on Earth and host more than half of all living species on the planet [90,91,92,93,94]. Some of the plants that grow there could have potential medicinal properties, including some that have not yet been discovered [95,96,97].

However, subtropical forests are located at lower latitudes than tropical forests and serve as transition zones between tropical and temperate climates [98]. These forests exhibit a more marked seasonality compared to the previous ones, with very well-defined wet and dry seasons [99,100,101]. Subtropical forests’ biodiversity is rich and varied, although not as extensive as tropical forests, and they are ecosystems that host a mixture of tropical and temperate species, giving them unique diversity [58,102]. These forests are crucial for buffering the impacts of climate change and improving the stability of ecosystem productivity over time [103,104].

The dominant ectomycorrhizal fungi in most subtropical forests generally have a low host specificity, although there is an exception to this pattern, which is the symbiosis with the genus Alnus [105]. It has been shown that the composition of some communities of these ectomycorrhizal fungi associated with trees of the *Alnus rhombifolia* species, in subtropical forests, are capable of generating defense against soils with high levels of heavy metals that are highly toxic to trees and their biodiversity [105,106,107]. This symbiotic effect can be attributed to alterations in soil conditions due to leaf litter and root exudates, which in turn play a crucial role in soil protection and carbon storage [108]. Subtropical forests, especially in southern and eastern China, are recognized for their wide diversity of root-associated plants and fungi, which contribute significantly to global ecosystem services such as carbon cycling and primary production [109,110]. 

Finally, we find boreal forests, also known as taiga forests, which stand out for their resistance and ability to adapt to challenging and extreme environments with a combination of long and very cold winters, with very short growing summers [111,112,113]. The boundary of the circumpolar biome at the interface of boreal forest (taiga) and tundra is a very important ecological transition zone for the planet [114]. They are characterized by a large extension of conifers, preferably of needle-like and broad-leaved deciduous types, capable of supporting cold climates and growing in short seasons. Evergreen coniferous boreal forests have a remarkable ability to acclimate to extremely cold temperatures, which is crucial for their survival in polar regions [115]. However, the same authors suggest that this forest type (mostly composed of conifers) is highly vulnerable to climate change, but further research is needed to understand and somehow mitigate these risks [112,116,117]. These forests are found in the northern regions of North America, Europe, and Asia [118]. The same authors state that biodiversity in boreal forests is less varied compared to the tropics and subtropics, but these forests are crucial for species specifically adapted to extremely cold climates with the presence of unique large mammals and numerous species of birds, fish, and insects. The loss of boreal forests can lead to habitat fragmentation, the displacement of native species, and the disruption of ecological relationships adapted to extreme climates [119]. Taiga is essential for global carbon storage and, at the same time, acts as an important climate regulator [120].

The loss of large tracts of native forests could have dramatic consequences for their immediate environment, altering the intricate and complex ecological interactions on which the living things that inhabit them depend and possibly even impact the carbon cycle [121,122]. The loss of native forests has significant negative consequences for the planet, especially in terms of population and biodiversity changes [123]. This loss can provoke positive and negative responses since changes at the local scale are intensified by up to 48% after forest loss. Additionally, the risk of species being listed as threatened or experiencing population declines increases substantially with deforestation, especially in the wilderness [121,122,124,125]. Selective tree retention practices would be important to promote biodiversity and reshaping boreal forest landscapes [126,127]. Tropical forests have significant implications for human well-being and the achievement of Sustainable Development Goals [128]. This is due to the interconnection of forest fauna and tree species with other plants, animals, and fungi, as well as their role in ecosystem functions and services [129]. The combined effect of biotic and abiotic stressors, exacerbated by climate change, further contributes to forest loss and a decline in biodiversity [130]. The loss of large species, especially vulnerable to extinction, may result in fewer interlinked food webs and reduced ecosystem functionality [92,121,122]. Table 2 shows the summary of the geographical distribution of the main countries with tropical, subtropical, and boreal forests. In addition, we can observe a summary of the different aspects related to climate, vegetation, diversity, impacts on biodiversity, carbon storage, and finally, the importance of the ecosystem of the three geographical forest regions analyzed.

### 2.2. Forest Degradation versus Extractive Deforestation and Impact on Biodiversity

In the context of global forest dynamics, it is imperative to differentiate the contrasting mechanisms related to two fundamental concepts, namely deforestation and forest degradation [131]. However, it should be noted that there is no scientific consensus on these two notions. There are many overlapping definitions, which complicates the picture when making comparisons between various studies and between countries, regions, and different types of forests [132,133,134]. However, forest loss is characterized by a combination of deforestation and forest degradation. Deforestation, according to our definition, is the permanent transformation over time of forest land, generally intending to give it other uses (agriculture, livestock, urban development, logging, mining, and oil), directly impacting the loss of biodiversity, erosion, and producing climatic changes. That is, deforestation refers to the abrupt change from trees-covered land to tree-free lands without any probability of future growth [135,136]. This characteristic refers to the complete conversion of forests to alternative land uses, leading to the complete loss of forest and ground cover [137]. 

**Figure 1:**
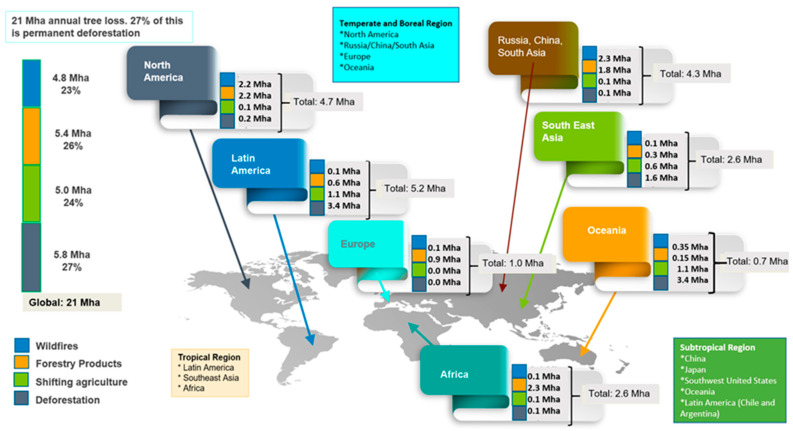
Global forest loss: deforestation versus forest degradation.

However, forest degradation is broadly understood as the disturbance caused by human or natural actions in a forest landscape that results in a reduction in the forest’s ability to provide goods and services [138]. Therefore, forest degradation refers to the temporary reduction in tree density in a given area, that is, the thinning of vegetation cover and the depletion of carbon content without altering land use, foreseeing a forest regrowth process in a peremptory time [136]. That is, forest degradation encapsulates the accelerated deterioration of the quality and ecological vitality of existing forest ecosystems, often due to factors such as unsustainable logging, pollution, insect attacks, arson, accidental fires, or changes in the global climate [138]. Forest degradation involves more subtle erosion, but also impacts the inherent health and functionality of forests over time through potential resilience [10,60,139,140,141,142]. From these elements, it is inferred that the environmental impacts of deforestation are more severe and permanent than the degradation produced by the substantial loss of forests in tropical, subtropical, and boreal geographic regions [143]. In this context, Figure 1 highlights some facts related to the loss of forests around the world related to deforestation versus forest degradation. In this case, we can observe the geographical region of tropical forests consisting of countries in Latin America, Southeast Asia, and Africa. Temperate/boreal regions include North America (the United States and Canada), Russia, China, South Asia, Northern Europe, and Southern Oceania. Finally, the subtropical forest region includes southern China, Japan, the southwestern United States, Oceania, southern Chile, and Argentina. Figure 1 also illustrates the loss of forests due to massive deforestation caused by human intervention (extractivism). At the global level, 27% of the 21 million hectares (Mha) of forest lost annually correspond to permanent deforestation, that is, to an irreversible change in land use [136]. This is key because deforested soils can hardly be recovered (due to the elimination of layers of fertile soil composed of networks of ectomycete fungi, rhizomes, and minerals, among other things) [144], while forests that have been degraded have higher chances of recovery in the medium and long term with a greater probability of resilience [145,146]. However, the tropical forest is the one that suffers the greatest combined deforestation and degradation on the planet, while at the same time, it is the richest in biodiversity and ecological complexity, with its loss negatively impacting not only the forest but also the global climate [136].

Continuing with Figure 1, the greatest pressure on these forests (the deforestation rate is observed in dark gray in the left column) is generated by human intervention since agriculture is the main factor responsible for its deforestation, with around 3.4 Mha of annual loss in Latin America alone, 1.6 Mha of loss in Southeast Asia, and finally, a loss of 0.1 Mha in Africa. Forest losses due to degradation in these same tropical forests are not minor, reaching 34% of the total loss. Regarding boreal/temperate forests, the greatest degradation is caused by forest fires and timber plantations (monocultures) to replace the native forest and its diversity for commercial purposes, which is equivalent to a significant 66% of total forest degradation, but that, nevertheless, barely reaches a loss of 5% of the global total due to deforestation [136,147]. Finally, in the subtropical forest region, the richness of tree species that coexist within it is extremely diverse, marked by the different seasons, so the variability in terms of niches and competitive capacities is evident. There is less precision about the volumes of degradation and deforestation in these forests. The largest number of studies related to them belong to research carried out in China and some in Latin America (Paraguay, Argentina, Chile, and Bolivia) [148]. As for Latin America, large areas of its forests have suffered a large amount of systematic deforestation, mainly due to the abusive use of agriculture (mainly pastures for livestock and soybean cultivation) and logging [149]. However, some temperate and subtropical forests have begun to have positive indices by increasing their forest cover, especially in central and southern China, where there is a good correlation between public policies for the implementation of ecological restoration projects and the rapid economic development of this country [150]. On the other hand, forest losses due to fires (accused and natural) in different geographic forest regions drastically change these relationships. Boreal forests have the highest proportion of fire losses, reaching 73%. They are followed by subtropical forests that lose between 19 and 22%, then temperate forests with losses between 17 and 21%, and finally, tropical forests that reach between 6 and 9% [151].

### 2.3. The Importance of Natural Capital versus Extractivism-Leading to Deforestation

Natural capital is gaining importance and visibility in measuring the economic performance and socio-cultural prosperity of different emerging economies [152]. Natural capital can be defined as the stock of natural resources that includes both land and ecosystems [57]. It occupies a more intrinsic rung of capital compared to anthropogenic constructs such as human capital, social capital, manufactured capital, and financial capital [153]. This distinction comes from its role in providing fundamental requirements for human existence, including biodiversity, livelihoods, and access to clean fresh water and air, as well as indispensable and crucial resources for the functioning of society [154].

From this perspective, extractive industries and natural capital play critical roles. However, it should be noted that extractive industries have historically been focused on three large conglomerates: oil, gas, and mining [155]. An important reflection on this definition leaves a large gap in the literature when it comes to deforestation, as it is largely absent in this trilogy, ignoring the enormous impact it has on the environment [154]. Deforestation as an extractivist phenomenon affects all niches related to natural capital and cascades to all biodiversity [156]. It is important to note that deforestation in general occurs for different reasons. The three most important purposes are related to the exploitation of mining, gas, and oil exploitation; the generation of land for agriculture and livestock; and finally, the construction sector [157,158,159,160,161,162].

Extractivism involves destructive practices that subjugate and deplete natural resources, degrade soil, accelerate species extinction, cause the decline of biological diversity, and destroy forests [66]. All cases are intrinsically related to the pursuit of anthropogenic capital accumulation and the constant drive toward exponential economic growth throughout the world [163]. In parallel, the increase in socioeconomic disparities at the global level in numerous geographic regions is another characteristic that usually drives the phenomenon of extractivism [164,165]. However, extractivism, if measured in the very short term from a socio-economic perspective, could generate a sensation of sometimes alleviating poverty, inequality, and unemployment in emerging countries because it generates financial resources quickly, but without being sustainable over time. However, if measured in the medium and long term, the environmental, social, cultural, and economic impact is generally negative due to the footprint it leaves on the intervened forest ecosystems [166]. At the same time, there is a lack of reciprocity in this practice, as it does not seek to protect these resources and, therefore, opposes sustainability [167]. The effects of extractivism, such as resource depletion, environmental degradation, and socioeconomic inequalities, are often associated with excessive capital accumulation and economic growth of some strong economic groups to the detriment of local communities [168]. These aspects form a systemic context known as disorganized development. Smart [166] analyzed extractivism from a conceptual theoretical perspective toward organizational development aligned with an ethical–political approach of ‘transformative global studies’. One could argue that there has been a shift towards an intensification of extractivism on a global scale [66]. Forest loss alters habitats and niches that support a diverse range of flora and fauna. Above-ground biodiversity, which includes plants, animals, and insects, depends on the forest canopy for shelter, sustenance, and breeding grounds. Deforestation in any of the geographical regions addressed (tropical, subtropical, or boreal) fragments this intricate vertical structure, displacing species and altering trophic interactions and the collective resilience of forest ecosystems [169,170].

### 2.4. Resilience in Tropical, Subtropical, and Boreal Forest Ecosystems

Resilience in forest ecosystems related to tropical, subtropical, and boreal forests is closely related to another concept known as hysteresis, which refers to the dependence of the current state of an ecosystem on its history and is a complementary indicator of forest resilience [171]. The definition of resilience is complex due to the varied interpretations that the literature makes depending on the discipline that addresses it [16,172]. This ambiguity is due in part to the extensive use of the term in different contexts. Scholars disagree on whether resilience is a system property, a process, or a management outcome [169,171,173,174,175]. However, three main concepts of resilience emerge in the literature: engineering resilience, ecological resilience, and socioecological resilience [57,176]. Nikinmaa [169] elaborates on the three models, where engineering resilience refers to the system response associated with rebuilding following a disturbance or catastrophe, quantified as the time it takes for variables to return to pre-equilibrium of the disturbance. Ecological resilience measures the persistence of the system and its ability to absorb change while preserving different relationships. Finally, socioecological resilience considers that natural and social systems are interconnected and focuses on general adaptive capacity. Furthermore, there is what is known as ‘The Alliance for Resilience’ [177], which characterizes resilience as the ability of a socio-ecological system to withstand disturbances, maintaining its structure and functions. This emphasizes self-organization, learning, and adaptation. Understanding resilience helps navigate the changing environmental and social dynamics for sustainable management of diverse ecosystems [169]. Resilience can be classified as resilient (i.e., no change after impact) or recovery (i.e., returning to the pre-impact state, but considering the development of management options that maintain both ecosystem services and human well-being) [178], which makes it somewhat similar to hysteresis.

### 2.5. The ‘Mother Tree’ Is the Center of the Resilience of the Ecosystem

Suzanne Simard’s research indirectly points to the importance of natural capital by noting that the interconnectedness of underground forests fosters mutualistic relationships that improve species diversity and resilience [11]. However, it is important to note that the research developed by Simard is the result of an evolution in the scientific understanding that preceded her on the different forest ecosystems [179,180]. In forestry, there is still a tendency to focus on models of stand dynamics driven by competition between trees and plants for limited resources such as light, water, and nutrients [181]. However, this perspective is beginning to change with the deepening of research in plant ecology and, particularly, in mycology [182]. One of the first advances was the recognition of the importance of the role of mycorrhizae and the symbiotic associations between fungi and plant roots with implications on the health and growth of trees in the forest [183,184,185]. This idea was initially explored by scientists such as Franciszek Kamienski and Albert Bernard Frank in the late 19th and early 20th centuries, who studied plant–fungus associations [186]. Later, researchers such as John L. Harper in the 1960s and 1970s provided a theoretical framework for understanding plant population dynamics, including competition and cooperation [187]. These studies began to suggest that plants not only competed with each other but could also cooperate in more complex ways [188]. Subsequently, forest ecology began to integrate these concepts, broadening the understanding of how mycorrhizae influenced plant–plant and plant–environment interactions and began to dig deeper from the further input of molecular biology [189]. Bonfante [190] provides a different perspective on mycorrhizal research, highlighting how past discoveries and hypotheses have formed the basis for the current understanding of plant–fungus interactions, and how these interactions would be fundamental to the health and sustainability of tropical, subtropical, and boreal forests as a whole. According to some authors, mycorrhizal networks would not only help plants absorb nutrients but could also facilitate the transfer of resources between different plants [191,192,193,194]. It could be speculated that these concepts, which are shared by some researchers, would be close to the line of research that Simard applied in some forests on the integration of these concepts based on plant–mycorrhizal interactions [17,21,22,25,195,196,197,198,199,200,201,202,203,204,205,206]. These investigations show a complex subway communication network. In this line, Simard estimates that mother trees would play a crucial role in maintaining the health and stability of the forest ecosystem by transferring nutrients and some signals to other younger and possibly more vulnerable trees in the environment. This mycorrhizal network, which Beiler et al. [207] described as the ‘Wood Wide Web’, would function as an interconnected and even cooperative system, challenging the traditional notion of fierce competition as the sole driver of forest dynamics [11,16].

The concept of “mother trees” understood from the perspective of older trees, with long-range roots and protective functions, emerges as a phenomenon of analysis in forest ecology, although controversial [11,26,205,208]. These trees (always according to Simard’s hypothesis) would be fundamental entities for maintaining forest health, biodiversity, and ecological stability. The mother trees, often characterized by their advanced age, larger size, and extensive root systems, would play a crucial role in shaping the intricate web of interactions that sustain forest ecosystems. The essence of mother trees would be their multifaceted relationships with neighboring trees [209]. Simard and other researchers suggest that through complex networks of root connections, these older trees could function as “nurturing” centers, extending resources and protection to their younger counterparts as a cooperative alternative [210,211]. This process would be mediated by certain mechanisms, including the transfer of essential nutrients, carbon compounds, and even defense-related molecules [11,18,212]. Through this symbiotic exchange of resources, it would not only sustain the growth of individual trees but also strengthen the collective resilience of the forest ecosystem as a whole [169]. One of the fundamental contributions of mother trees to ecosystem resilience, still according to Simard’s research, would be their role in transmitting resources in times of stress. Because these mother trees would apparently possess a large number of resources accumulated over decades of growth, they would be in a position to mitigate the impacts of various stressors, such as drought, disease, or nutrient shortages at any given time, both for themselves and for more vulnerable trees in the environment [213]. If so, this role could become especially crucial in the face of environmental fluctuations or disturbances, where the availability of resources could be limited for younger trees. Carreón-Ortiz and Valdez [214] support the importance of mother trees as reservoirs of resources that contribute to the survival and adaptive capacity of the forest community in the face of adverse conditions. Following these lines of research from a resilience perspective, it could be pointed out that the so-called mother trees, with their complex interaction with mycorrhizae, would play an important cooperative role since they could contribute to the maintenance of biodiversity and nutrient, carbon, and water cycles, essential for the health of ecosystems [11]. In tropical forests, resilience is revealed as the ability of these ecosystems to adapt and recover from events such as deforestation or abrupt and seasonal changes in the rainfall regime [215,216]. Meanwhile, in subtropical forests, resilience is crucial to the rapid adaptation of trees to seasonal variations in rainfall and temperatures and to the progressive changes evident in climate [217,218]. On the other hand, in boreal forests, resilience is observed in their ability to regenerate after natural disturbances, which are, most of the time, generated by forest fires and insects in a highly adverse climate [111,219]. Despite extreme climatic conditions in boreal forests and nutrient-poor soils, the symbiotic relationships between mature trees and mycorrhizae ensure the survival and regeneration of the ecosystem [208]. This resilience is a clear example of resilience and its ability to face and adapt to constant disturbances over different periods.

According to Simard’s research, mother trees would be dominant individuals in the forest, able to share their energy sources with other trees with less access to sunlight, water, and other nutrients. Simard estimates that many seedlings when they first germinate in the understory are colonized by a network of mycorrhizae and begin to receive not only nutrients from the soil but also carbohydrates from these established trees as a nurse effect, where they would be protected from herbivores, organic matter, and even defense signals [208,220].

### 2.6. The Underground Kingdom of Mycorrhizal Networks Responsible for Forest Biodiversity

Mycorrhizae constitute a crucial component of one of the most extensive and vital biological interactions between different kingdoms, connecting more than 340,000 species of terrestrial plants with around 50,000 taxa of soil fungi [221]. This intricate subterranean kingdom of mycorrhizal plants lies beneath the forest floor, where trees and fungi establish symbiotic relationships that facilitate the exchange of nutrients and vital information [208]. Suzanne Simard’s research has revealed the impact of deforestation on the tangled functioning of these networks, underscoring their role in enabling resource exchange mechanisms between trees and fostering mutual support [222]. Deforestation caused by extractive practices affects not only trees and plants but also mycorrhizal networks and a set of complex connections (a network of interactions between plants and microorganisms) that impact critical ecological processes [223,224]. The loss of forest areas (tropical, subtropical, and boreal) could lead to the breakdown of these intricate connections, which, in turn, would affect critical ecological processes such as nutrient cycling, soil structure and stability, and the complex network of symbiotic interactions between plants and microorganisms [11,208]. Consequently, this alteration could have an impact on ecosystems, influencing the health of plants, biodiversity, and the general functioning of the environment. Mycorrhizal networks represent a fundamental link of interaction between plants and fungi, forming the backbone of intricate ecological systems [225]. Simard’s research emphasizes the interconnection of these networks with the health and resilience of forests and their wider impact on terrestrial ecosystems [208].

Recognizing the importance of mycorrhizal fungi/plant interactions is essential for the informed management and conservation of native forest landscapes and the myriad benefits they confer to both the environment and society. Therefore, it is important to address some specific varieties of mycorrhizal fungi and their main physiological needs depending on whether they are present in tropical, subtropical, or boreal forests and their adaptation to the plants and trees with which they interact and function in each ecological environment [226,227].

In tropical forests, arbuscular mycorrhizae are common and include genera such as Glomus, Acaulospora, and Scutellospora [228,229]. Regarding their physiological needs, these fungi adapt to conditions of high humidity and very warm temperatures [230]. They need soils in which they can exchange nutrients with a wide variety of plants [231]. In addition, they are crucial to facilitate the absorption of phosphorus and other nutrients in soils that are often very poor in organic matter [232].

Subtropical forests are characterized by a mixture of ectomycorrhiza and endomycorrhizas. Ectomycorrhizas include genera such as Pisolithus and Laccaria, while arbuscular endomycorrhizas are also present [233,234]. Ectomycorrhizae in these forests adapt to more variable conditions, including dry and wet seasons [235]. Therefore, these fungi are important for the absorption of nutrients in soils with alternate periods of drought and humidity [236]. Endomycorrhizas play an important role in the assimilation of nutrients, especially in plants that do not form ectomycorrhizas [237].

Finally, in boreal forests, the predominant fungal varieties are ectomycorrhizas, with genera such as Russula, Amanita, and Suillus [238]. These fungi form associations mainly with coniferous trees such as pines and firs [219]. The ectomycorrhizas in these forests are adapted to acid soils and cold climates. They require the ability to survive and function efficiently under low-temperature conditions, often frozen by permafrost, and in soils with low biological activity precisely due to cold [239]. However, these fungi achieve their goal by helping trees access nutrients in an environment where the decomposition of organic material is slow and the availability of nutrients is limited in time [240].

#### Molecular Studies on Mycorrhizal Fungi in Forests

In the field of forest ecology, molecular research on arbuscular mycorrhizal fungi has gained crucial importance through the use of biotechnology and genetic engineering [241]. These studies have provided a deeper understanding of the symbiotic interactions between trees and fungi, which is essential to better understanding the dynamics of forest ecosystems. Miyauchi et al. [242] have been pioneers in the field of mycorrhizal fungal genomics, providing significant information on the early evolution of the symbiotic traits of these organisms. Through large-scale sequencing, the same authors have brought to light key elements of how these symbiotic interactions have developed and how they work. On the other hand, Shi et al. [243] have examined variations in fungal communities along disturbance gradients in forests, showing how changes in the environment, both natural and anthropogenic, affect mycorrhizal networks and, consequently, the health of the forest ecosystem. This research highlights the sensitivity of mycorrhizae to disturbances and their vital role in ecosystem stability. From a molecular perspective, the interactive roles of different types of fungi in tropical, subtropical, and boreal forest ecosystems have been confirmed by genetic markers [244]. These advances have allowed us to observe in more detail the factors that influence the distribution of mycorrhizal and soil fungi, explore how these organisms interact with their environment, and provide valuable information for the proper management of forest needs [245]. Research on mycorrhizal interactions in orchids, ectomycorrhizal fungi, and ericoids has provided valuable insight into the molecular mechanisms of these symbioses. Studies have identified specific genes and pathways involved in these interactions, such as those related to nitrogen uptake and symbiotic marker genes [246]. Other genomic studies have identified key genes involved in the uptake and symbiosis development, shedding light on the complex dynamics of mycorrhizal associations [247].

In the context of climate change, some studies show how mycorrhizal symbiosis can improve the adaptation of trees to different types of abiotic stress, which is vital for the resilience of forests, mainly temperate and boreal, in the face of environmental changes. [207]. Advances in the functional roles of fungal endophytes in the microbiomes of forest trees have provided a comprehensive view of the interaction between trees and their fungal symbionts that impact forest health [248]. This study highlights the importance of endophytes in the regulation of ecosystem functions and the promotion of forest health. On the other hand, a direct connection has been established between forest tree growth and the composition and function of mycorrhizal fungi [194]. Shi et al. [249] analyzed the impact of deforestation on the soils of fungal diversity and community composition in a tropical rainforest. They found that the richness of saprotrophic soil fungi, including phosphorus-solubilizing fungi such as Penicillium spp., decreased as forest disturbance increased, while facultative pathogenic fungi became more abundant. This change suggests a transition from phosphorus limitation in undisturbed forests to carbon limitation in deforested areas, highlighting a potential obstacle to plant succession after deforestation. The study highlights the sensitivity of soil fungi to forest disturbances, indicating their potential as indicators of soil health and the interaction between above-ground and below-ground ecosystems. In the network of symbiotic interactions between plants and microorganisms, the former act as hosts for a wide range of microorganisms from their environment or inherited from parental sources [250]. This mutual commitment extends to specific bacteria that play a critical role in the support of plant health and growth [251]. Among these bacteria, certain strains offer the unique ability to fix nitrogen, a critical process for enriching the soil with this essential nutrient, which is then taken up by higher plants [248]. Furthermore, these microorganisms influence the regulation of plant hormones, which further contributes to the balance of plant physiology [250].

However, the symbiotic harmony of these processes may be threatened by deforestation generated by extractive practices. The ramifying consequences of forest clearance extend beyond the visible landscape and reverberate through these subway pathways, potentially altering nutrient dynamics and compromising the very basis of forest vitality [252,253]. Soil fertility would be compromised as the nutrient exchange network is dismantled [254]. The finely tuned balance of the nitrogen cycle would be out of balance, resulting in the breakdown of essential biogeochemical processes that underpin ecosystem functionality [39,255]. In essence, the interaction of plants, microorganisms, and their sophisticated connections highlights the important harmony within forest ecosystems, but again, the phenomenon of coopetition comes into play in the whole organization of forest biodiversity [40]. All these studies provide a stronger scientific basis for the importance of mycorrhizal fungi in forest ecosystems and may provide some clues at the same time to Suzanne Simard’s hypothesis on the dependence of arboreal communication on mycorrhizal fungi. Molecular research in this field not only broadens our understanding of forest ecology but also opens new avenues for the conservation and sustainable management of forest ecosystems and the deepening of resilience and the impact of potential ecological cascading effects.

### 2.7. Cascading Ecological Effects

The ramifications of the effects of deforestation extend far beyond the confines of the forest itself, regardless of whether it is tropical, subtropical, or boreal. They encompass, as we have seen, a complex series of ‘cascading’ ecological effects that impact interconnected ecosystems and, ultimately, forest biodiversity [256]. A profound transformation in land use triggers a chain of repercussions that extends across several environmental compartments, with significant implications for both terrestrial and aquatic domains [257]. One of the main ecological cascades derived from deforestation involves alterations in the dynamics of nutrient cycling [258]. Through different investigations, it has been established that intensive deforestation would have cascading ecological effects, affecting tree growth, carbon assimilation, and concentrations of nonstructural carbohydrates [224,259,260,261,262]. As forests are cleared, the intricate cycle of nutrient exchange that characterizes these ecosystems is disrupted [263]. Organic matter, often rich in nutrients, present in the form of leaf litter, fallen branches, and decaying vegetation, mainly in subtropical forests, plays a fundamental role in soil nutrition and provides essential elements for plant growth [264]. With deforestation, this cycle is broken and the flow of nutrients from vegetation to soil and waterways is affected [265]. This, in turn, decreases soil fertility and causes a reduction in primary productivity, ultimately influencing the composition and structure of the surrounding ecosystems, an element very often seen in tropical forests [266]. Furthermore, deforestation exerts a perceptible impact on hydrological processes [267]. Intact forest cover intercepts rain, slowing its descent to the forest floor. This allows the soil to gradually absorb water and subsequently release it, maintaining a constant flow in local surface and underground waterways. Deforestation disrupts this intricate mechanism, accelerating the flow of rainwater into the soil [268]. Consequently, the ability of the soil to absorb water is compromised, resulting in reduced water retention and increased surface runoff [269]. This leads to increased soil erosion, as sediment-laden runoff washes away the land and is deposited in adjacent water bodies [265]. The alteration of hydrological cycles extends its influence downstream, affecting adjacent waterways and the communities that depend on them [270]. Irregular water flow patterns, altered sediment transport, and changes in water quality can have profound effects on aquatic ecosystems, from the alteration of fish habitats to the degradation of water sources that human populations depend on [271].

The notion of ‘cascading’ ecological effects emphasizes the deep interconnectedness of ecosystems and the intricate interaction of ecological processes. The impacts of deforestation are great, but the effects of reforestation could also be harmful due to the compaction of their soils due to the passage of the heavy machinery used [272,273]. However, it should be noted that not all forest intervention strategies for restoration purposes can be categorized as good or bad, as they depend on numerous variables. In some cases, natural restoration may be the most efficient in recovering much of the ecological services lost through the degradation of some tropical forests, while in others, this may not be the case [223,274,275]. Shimamoto et al. [274] conducted a global meta-analysis of ecological indicators of ecological services provided in restored areas, degraded areas, and reference ecosystems of tropical forests where restoration strategies recovered ecological services to varying degrees, and they reported that in practically all cases, there were positive effects. Some research has reported on the complex relationship between forest extractivism and its relationship with mycorrhizal fungi, suggesting that some stressors, such as deforestation, can cause the loss of the mycorrhizal community, which in turn can lead to tree decline in the form of a vicious cascading cycle [260,261]. In some cases, a virulent attack was evident in some plant species in which disturbance of the forest was observed to alter the composition of the community of ectomycorrhizal fungi and arbuscular mycorrhizae with a lower richness and diversity than in areas that had not suffered disturbance [262].

Beyond the mere loss of forest cover, they resonate through intricate nutrient cycles and hydrological dynamics, affecting not only the immediate environment but also extending its consequences downstream and in the vadose zone [8,276]. Recognizing and understanding these cascading effects is essential to designing effective public intervention strategies for conservation and land-use management that fully consider the long-term consequences of extractive human activities on natural systems.

### 2.8. Importance of Public Policies, Institutions, and Governance in the Regulation of Extractivism for a More Sustainable Development

**Figure 2:**
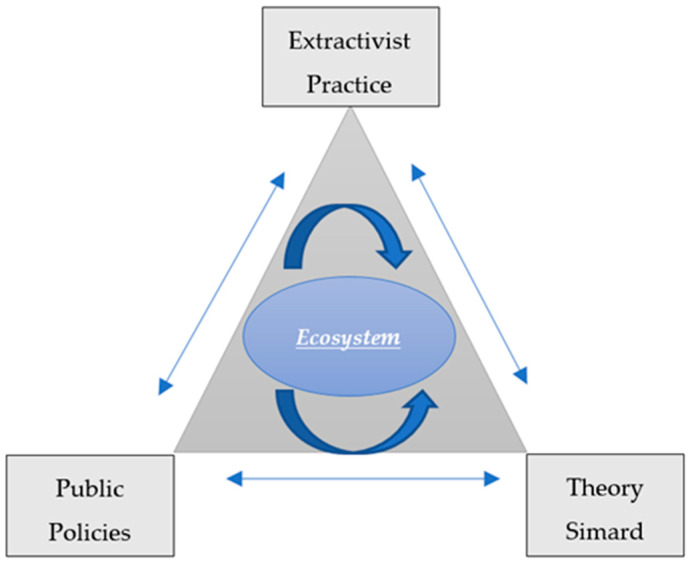
Interrelationship between the different ‘external’ actors that guarantee the survival of forest biodiversity.

Developing countries are often characterized by a confluence of factors that hinder the protection of natural resources and the sustainable management of ecosystems in the form of natural capital [153,277,278]. In these countries, an important challenge is forest management of the overexploitation as a result of extractive practices by large foreign corporations that, in many cases, act with total irresponsibility in the face of regulatory gaps that limit their predatory actions [154]. Institutions and governance, coupled with poor policies and corruption phenomena, create an environment conducive to the vilification of existing regulations, which generally leads to unprecedented ecological degradation [57]. Figure 2 shows the influence that the actions of different ‘external’ agents have on natural ecosystems. Each vertex of the triangle influences and dynamically impacts the whole with its action or inaction, particularly the vertices referring to public policies and extractivist practices.

#### 2.8.1. Poor Public Policies, Governance, and Institutional Frameworks

Public policies play a critical role in shaping the regulatory landscape for the extraction of natural resources [279]. However, many developing countries struggle with inadequately formulated policies that do not adequately address environmental concerns and local social interests [280,281,282]. These policies often lack clarity, enforceability, and provisions to curb overexploitation. Furthermore, institutional frameworks designed to supervise these policies are often poorly equipped, with limited resources, technical expertise, and enforcement capabilities [246]. Corruption within governance compounds the problem by undermining regulatory efforts [282]. When regulations can be circumvented through bribery or other illicit means, many large extractive corporations exploit this vulnerability, aggravating forest degradation [283].

#### 2.8.2. Exploitative Extractivist Practices by Multinational Corporations and Biodiversity Loss

Large multinational corporations often take advantage of weak governance systems and institutional inefficiencies to carry out extractivist practices with minimal regard for ecological sustainability [284]. These practices include indiscriminate logging, open-pit mining, agriculture, and urbanization, leading to massive deforestation and loss of biodiversity [285]. The lack of effective enforcement mechanisms allows these companies to operate beyond the limits of sustainable development [286]. The consequences of overexploitation are dire and lead to the degradation of native forests and the loss of tree cover [287]. The intricate biodiversity that thrives in these ecosystems is in danger, as habitats are destroyed and animal species lose their refuges and plants their substrates [288] without losing sight of the importance of the resilience of socioecological systems [31].

## 3. Discussion

Geographic regions belonging to tropical, subtropical, and boreal forests constitute some of the ecosystems that most significantly impact the quality of the biosphere of our planet [50,78,289]. Therefore, the different disturbances generated by extractive deforestation carried out in these forests would reveal a complex confluence of factors that go beyond the purely ecological and involve socioeconomic and political agents that demand adequate sustainable management of forest practices [211,290]. In this line, the discussion addresses two essential elements. The first is the importance given to the different coopetition networks that exist between mother trees, their environment, and mycorrhizal fungi. In this context, different conceptual positions in research have contributed to the debate in the scientific community and have not yet been settled [11,16,18,20,21,22,23,24,25,26,27,66,121,122,123,126,127,163,191,192,193,194,195,208,209,212,213,214,220,237]. Some authors estimate that the conservation of these mycorrhizal networks could have a direct impact on the preservation of the health and resilience of forests of the geographic regions addressed [11,26,187,193,291], while others estimate that they are organisms that would act differently depending on the needs and stresses to which they are subjected in the ecosystem [26,27,220]. The second element of the discussion refers to the possible environmental consequences of extractive practices related to deforestation in relation to other less sustainable industries such as agriculture and particularly oil or mining [70,155,211,290]. A specific case of mining extractivism can be observed in the tropical forest geographical region in the Amazon with dire consequences for the surrounding forest ecosystem, affecting its fragile soils, in addition to transporting potential heavy metals to the vadose zone due to water runoff [292,293,294]. In the absence of conclusive scientific evidence, this review invites us to reflect on how the adaptation of best anthropogenic practices can be aligned with natural processes, ensuring more sustainable development, both for forested geographic regions and for humanity in general. All this is from the lessons obtained from hysteresis and resilience to improve our interpretation of the changing dynamics of these intervened ecosystems [171,295,296,297].

Simard’s research has attracted both supporters and detractors among scientists. Still, it is the context-dependency of the results and their extrapolation beyond the systems investigated that drives the controversy. Research that is more aligned with Simard’s hypothesis suggests that some functions of the roots of the mother trees generate a mutual dependence on the mycorrhizal community, which would provide an advantageous resource to evaluate the complexity of forest ecosystems when they are intervened, for example, anthropogenically [191,192,193,194,298]. Other scientists consider this quasi-thoughtful position controversial and therefore a resisted hypothesis since they estimate that mycorrhizal fungal communities could respond almost autonomously to the environment in which they are found, beyond the complex ecosystem of mother trees in which they may be found, and therefore question the mother tree hypothesis, suggesting that evidence of significant transfer of carbon through mycorrhizal networks is lacking [27]. Similarly, other researchers opposing Simard’s hypothesis suggest the possible involvement of a “common symbiosis path” in the establishment and maintenance of ectomycorrhizal associations [224,299]. Other research analyzes the complex relationship between tree decay and mycorrhizal fungi from different perspectives.

In this quest to align this dichotomy of research currents, efforts are being made to identify the role of mycorrhizae in carbon and nitrogen sequestration and their interrelationship with tree roots and other organisms at the molecular level through genetic engineering techniques that should shed more light on this complex discussion [34,35,241,246,247]. Other researchers seek more moderation, as is the case of Marin and Bueno [300], who highlight the need for a more balanced approach to the investigation of mycorrhizae and their interactions. Meanwhile, other research estimates that the formation of mycorrhizal networks next to the roots of some plants not only affects the distribution of scarce nutrients, mainly nitrogen among interconnected plants, but is also vital for the growth of these while building a pathway for carbon assimilated by these plants [301,302,303]. From these mechanisms, we can appreciate the importance that coopetition would assume within the forest ecosystem, which suggests that competition and cooperation between trees of the same and different species can co-exist beneficially, facilitating a dynamic balance that would maintain diversity and ecosystem resilience [304,305]. In this sense, biodiversity is crucial for forest health, as many species would make unique contributions to ecosystem functioning and resilience and also compete with each other, confirming the phenomenon of coopetition. This is essential to understanding the functionality and interaction of these ecosystems based on the simultaneous occurrence of cooperation and competition among trees within the biosphere. While trees compete for sunlight, water, and nutrients, they also cooperate by exchanging essential resources through different subway root systems [306]. This coopetition approach could inspire new intervention, conservation, and sustainable forest management strategies that mimic and take advantage of these natural interactions and, at the same time, can serve as a tourist attraction with important socioeconomic benefits.

However, one of the critical points to highlight in this discussion is to reflect on the detrimental impact of deforestation driven by extractive practices that could transversally alter the delicate balance of forest ecosystems [150,307]. While the exploitation and use of natural resources are essential for human development and economic growth, the uncontrolled exploitation of these resources often leads to rapid environmental degradation, loss of biodiversity, and ultimately, socioeconomic disparities [2]. This is because often a forest sector is overexploited thinking that immediate economic benefits are achieved for local communities, but the consequences are usually disastrous in the long term since forests, soils, and water are extinct, generating more poverty and desertification [308,309]. The concept of extractivism, which is associated with the concept of extractive industries, is based on the accumulation of financial capital and rapid economic growth at any cost, leading to practices that disrupt the intricate relationships within forests and ultimately compromise their resilience and integrity in the medium and long term [66]. Deforestation disrupts the symbiotic relationship between trees and mycorrhizal fungi, crucial for nutrient exchange and plant growth, leading to a decline in forest biodiversity and productivity [310]. Then, it is evident that extractive deforestation should have devastating effects on biodiversity and ecosystem services provided by forests [311]. It is urgent that political institutions address this problem based on the importance of tropical, subtropical, and boreal forests and their complex ecological networks. To this end, the integration of scientific findings on mycorrhizal networks, together with stronger public policies and informed and sustainable forest management, is essential to preserve these vital forest ecosystems for the planet [11,35,184]. However, it is essential to go even deeper to better understand the interactions between plants, soil, and mycorrhizae, and for this, advances at the biotechnological level (molecular and genetic) can provide new approaches to understanding and improving forest health of forests and their ability to withstand environmental and anthropogenic pressures [241,242,243,244,245,312,313]. These new lines of molecular research could reveal innovative strategies for the restoration of damaged forests and the sustainable development of forests [241,242,243,244,245,246,247]. Increasing the capacity to resist stress in the forest ecosystem, adapting to changes in the environment, and recovering from potential disturbances of human or environmental origin are key to the success of a healthy forest that thrives over time [314]. The impacts of deforestation are not only limited to the visible landscape [315]. The interruption of underground networks, such as roots, that facilitate the exchange of nutrients and support interactions between plants and microorganisms has far-reaching consequences, in many cases, greater than what happens on the surface [316,317,318]. This alteration compromises soil fertility, biogeochemical processes, and essential ecological functions, affecting the vitality of the entire ecosystem and its biodiversity [319].

The loss of mother trees usually occurs because, being the most voluminous, they become the most coveted, mainly by the timber industry. The big problem is that when these specimens are removed, not only is species diversity reduced but there is also the risk of fundamentally interrupting the flow that could exist between mycorrhizal networks essential for the exchange of nutrients and signals between young trees of the same and different species and can drastically affect the rest of the forest ecosystem as a whole [211,223,292]. In some cases, an increase in arbuscular mycorrhizae as a consequence of anthropogenic intervention would have the potential to induce the acceleration of nutrients, with critical consequences for forest productivity, carbon availability, and nutrient retention [310]. The dissemination of Simard’s hypothesis on mother trees and mycorrhizal networks (although not yet categorically proven) still offers a preventive and precautionary approach to the sustainable development of natural capital through more responsible forest management [292]. Simard suggests that by protecting and conserving the hub of mature trees (mother trees) and their symbiotic mycorrhizal networks, the resilience and recovery capacity of forests improve when there is deforestation or forest degradation of younger trees [11,320,321]. This perspective highlights the need for a holistic approach to forest management, which recognizes the importance of complex biological interactions and coopetition within diverse forest ecosystems [174,184,322,323,324]. This brings us to the notion of ‘cascading’ ecological effects [325]. A broader perspective, from a hierarchical meta-analysis, reveals the complex interactions between global change factors and ecosystem function [326], with the synthesis of carbon cycling experiments underscoring the need for site-specific considerations in Earth system models [327]. These studies collectively underscore the multifaceted nature of deforestation and the importance of considering its ecological and economic impacts. Deforestation, whether in boreal, tropical, or subtropical forests, appears to trigger a chain reaction or a cascade of domino-style repercussions that could extend beyond the limits of the logged forests, likely affecting hydrological processes, nutrient cycles, and even aquatic ecosystems, negatively impacting even the vadose zone [224,260,261,295,328,329].

Regarding the effects generated by the ‘cascade’ chain, mention must also be made of the aspects related to the socioeconomic and public policies that impact the protection or not of a forest ecosystem. Busch et al. [330] provide evidence of the economic and environmental drivers of deforestation, highlighting the greatest exponent in agriculture. Similarly, Desbureaux and Damania [331] highlight the impact of agriculture as the main link within the cascading effects that accelerate the deforestation processes in many forests around the world. Therefore, it is important to generate the necessary conditions for sustainable forest development in the medium and long term, through coherent public policies, responsible institutions, and governance committed to society and natural capital [54,80,81,82,83,84,85,86,87,88,89,90,91,92,93,332,333,334,335,336,337,338], especially if one considers the lack of conclusive scientific unanimity regarding the functions of mycorrhizae and the relevance of the roots of the mother trees. 

Forest overexploitation has been especially promoted in developing countries, due to inadequate public policies, and is fueled in some cases by acts of corruption in their care, affecting the capacity of these ecosystems to act as carbon sinks and jeopardizing global efforts to mitigate climate change [339,340,341]. This means that strengthening public policies, institutions, and governance is crucial to more effectively protect forests in all geographic regions addressed until agreements are reached on the functions of the different components of the forest ecosystem [342,343]. This includes developing strategies that limit extractive exploitation and promote sustainable practices, as well as improving the monitoring and enforcement of existing global environmental regulations [311,344,345].

It is important to note that forests in different geographic regions have different capacities and requirements, but all are transcendentally important. Disturbances affect different ecosystems differently. Some plants and organisms accept disturbed environments well, while others tolerate them poorly, as is the case with mycorrhizal fungi [346]; however, tree decline might affect forest dynamics through plant–soil biota feedback [347]. Tropical forests, located in the equatorial regions of the planet, are a source of exceptional biodiversity [348]. They represent a vibrant mosaic of life and harbor more than half of the terrestrial species, although they cover only a small percentage of the total land area [92]. These forests play a crucial role in the regulation of the global climate and as sinks for enormous amounts of carbon [349]. The interconnectedness between species in these forests, as proposed by Simard’s hypothesis, means that the health of a single parent tree could significantly influence the surrounding forest community by seemingly creating a network of support and nourishment that could likely collaborate in ecosystem health [350,351]. On the other hand, subtropical forests, located at lower latitudes, act as transition zones between tropical and temperate regions [352]. These forests include a variety of ecosystem types, from humid forests to savannas and chaparral [353,354]. Although they do not have as much biodiversity as tropical forests, they are equally crucial to the conservation of numerous species and protection against soil erosion and carbon sequestration [58,92,355]. The importance of older and larger trees in these forests is fundamental, along with the centers of mycorrhizal networks, for the good condition and resilience of subtropical ecosystems [356]. Finally, vast boreal forests represent one of the largest biomes in the world, characterized by their cold climate and predominantly coniferous tree species that are very important in the generation of oxygen on the planet [118,357,358]. Boreal forests are vital for biodiversity, especially as a habitat for species adapted to cold climates and as an important carbon storage [359]. In these forests, Simard’s hypothesis could acquire a particular dimension since the extreme conditions of their climate and poor soils would mean that the interconnection and mutual support between trees through mycorrhizal networks could play an important role in the survival and prosperity of the ecosystem there [360,361].

## 4. Conclusions

This research shows the complex interaction of ecological, scientific, socioeconomic, and political factors that shape the notion of the phenomenon of extractivism and its potential consequences in the geographical forest regions analyzed.

There are fundamental aspects that emerge about the research questions and the objectives raised in this research. The controversial hypothesis about mother trees put forward by Suzanne Simard with her detractors and supporters stands out. Although Simard’s research has sparked lively debates within the international scientific community, it serves as a wake-up call to the pressing need to generate more knowledge that will allow for a greater understanding related to divergences in the functions of mycorrhizae and trees that make up forest ecosystems. The different scientific currents are far from reaching unanimous agreements on the functionality of mycorrhizal communities and the roots of mother trees, but also on the specific functionality of mycorrhizals and the way they capture carbon and nitrogen. Today, the knowledge gaps about the different postulates in these topics are very wide and there is no evidence that is conclusive or that solves the riddle about the potential impacts related to forest extractivism. However, there are efforts to be more optimistic about closing these gaps. Scientific research is increasingly focusing on the application of advanced genetic engineering techniques in mycorrhizae and the adoption of multidisciplinary research approaches that consider the multifaceted nature of forest ecosystems from a more resilient perspective. In this line, it is crucial to recognize that complexity is provided by the biological requirements and needs of each geographic forest region analyzed.

The concept of coopetition, in which trees compete for vital resources while cooperating across different underground, surface, and aerial networks, challenges conventional notions of competition-driven ecosystems. The coopetition is a generator of knowledge and learning that can allow us to generate more efficient holistic forest management strategies in the medium and long term. However, the threats posed by extractive practices pose a serious threat to forest biodiversity, soil fertility, and the resilience of the ecosystems involved, ultimately endangering the well-being of terrestrial and aquatic ecosystems. This leads us to suggest the urgent implementation of coherent public policies and responsible governance mechanisms to protect forests on a global scale. It is imperative to strengthen public institutions to address critical economic and environmental factors related to both deforestation and reforestation and to promote sustainable practices that protect forest ecosystems for future generations.

In essence, this research underscores the urgent need for a paradigm shift in our approach to forest conservation and management, regardless of the controversies surrounding both Simard’s hypothesis and the other research listed in this research. The aim is to foster a deeper interest and understanding of the intricate relationships within forest ecosystems and to foster the adoption of evidence-based practices. The ultimate objective is to reflect on the problem of forest extractivism and its still unknown consequences on different ecosystems and their biodiversity. In particular, in the absence of greater scientific knowledge, forests are often destroyed and altered irreversibly without quantifying irreparable damage in the future. Prudence should be the rule until more conclusive research results on the function and mode of operation of mycorrhizae and tree roots are available before further progress into the unknown. Even today, despite scientific advances at the molecular level and differences in the positions of researchers, we do not know the potential consequences of an anthropogenic intervention.

## 5. Future Directions

This theoretical framework becomes an invaluable tool for guiding future research and conservation policies, ensuring the preservation and sustainability of these forest ecosystems crucial to the health of our planet. This may include more detailed studies on symbiotic interactions, forest responses to deforestation by fire or pests, and the development of more sustainable management strategies. Building on the foundation established in this research, a deeper look into the complex interaction between deforestation caused by intentional, accidental, or natural fires and the intricate ecological dynamics within forest ecosystems is crucial. The same happens with the impact caused by the use of heavy machinery used in deforestation, but also frequently used in reforestation, compacting the delicate substrate and killing mycorrhizal networks. This avenue of research is of immense importance as it adds a layer of complexity to the already multifaceted challenges posed by indiscriminate deforestation and extractivism.

We must also investigate the synergistic effects of fire-induced deforestation compared to extractive activities because understanding how intentional or accidental fires differ or are not different from extractivism would provide valuable information on the cumulative effects of these different processes. Researchers could explore how fire alters nutrient cycling and mycorrhizal networks and influences the resilience of mother trees in deforested areas, combined with the benefits it brings to certain biodiversity. Additionally, studying how these combined stressors impact the survival and recovery of forest ecosystems would be essential to guiding effective public policy strategies for forest conservation and restoration. In other words, how do we generate a public policy that addresses extractive practices, ecological training, and forest protection? Furthermore, research could focus on the mechanisms through which natural fires shape forest dynamics in the context of extractive activities. Investigating how natural fire regimes impact the ecological dynamics of biodiversity in the face of extractivism-induced deforestation could reveal valuable insights into the complex feedback loops that influence post-fire recovery and ecosystem resilience. This would involve studying the role of fire-adapted species, the regenerative capacity of mother trees, and the subsequent establishment of mycorrhizal networks in fire-prone landscapes. Incorporating the influence of fire also raises questions and requires a comprehensive assessment of long-term effects on underground biodiversity, such as soil microbial communities and nutrient availability. Examining how fire shapes the functional traits of surviving trees and their connections to mother trees would provide a holistic understanding of ecosystem responses. This could involve implementing controlled burns, sustainable logging practices, and restoration efforts that encourage the recovery of mycorrhizal networks and the nutritional role of parent trees. Along the same line, the contributions of genetic engineering, biotechnology, and molecular research on mycorrhizal networks and the relationships with pre- and post-fire forests would help to understand their dynamics and the extent of the effect of fire in areas deeper underground to be able to act more promptly in the event of an incident of this type.

## Figures and Tables

**Table 1:** Analysis of environmental research keywords: frequency, contextual themes, and potential implications in forest ecosystem studies.

Keyword	Frequency (*n* = 1472)	Contextual Themes	Potential Implications
Mother trees: Guardians of ecosystem resilience	46	Ecosystem management, Forest resilience	Importance of key species in ecosystem recovery
The hypothesis of Suzanne Simard	33	Mycorrhizal networks, Inter-species communication	Foundation for understanding forest symbiotic relationships
Symbiotic relationships	55	Biodiversity, Ecosystem health	Role in ecosystem stability and resilience
Fungi, plants, carbohydrates, and water	51	Nutrient cycles, Water dynamics	Critical aspects of forest ecosystems’ functioning
Impact of deforestation, extraction and biodiversity	77	Deforestation impacts, Biodiversity loss	Effects on global biodiversity and climate
Underground biodiversity and mycorrhiza networks	49	Soil health, Mycorrhizae	Role of soil biodiversity in nutrient exchange
Arbuscular mycorrhizae	32	Plant-fungi interaction	Importance in plant nutrition and soil health
Endomycorrhizas and ectomycorrhizas	57	Types of mycorrhizal fungi	Differences in symbiosis and ecosystem roles
Mycorrhizae and genetic engineering	10	Biotechnology applications	Potential for enhancing plant resilience
Biotechnology and mycorrhizae	19	Biotech in ecosystem management	Innovations in sustainable forestry practices
Molecular research of mycorrhizal fungi	16	Scientific advancements	Contributions to understanding mycorrhizal functions
Nitrogen collection	34	Nitrogen cycle, Plant adaptation	Efficiency of Nitrogen Uptake and ecosystem nutrient cycling
Coopetition in the Forest	19	Inter-species relationships	Competitive and cooperative dynamics in forest ecosystems
Dynamic ecosystem and biodiversity	63	Ecosystem dynamics, Biodiversity importance	Impact of diversity on ecosystem resilience
Forest resilience	65	Response to disturbances	Strategies for enhancing forest recovery and sustainability
Resilience and disruption	47	Effects of environmental stressors	Mechanisms of ecosystem adaptation and survival
Healthy Forests	52	Indicators of forest health	Relationship with biodiversity and ecosystem services
Overexploitation and depletion of resources	68	Resource management	Consequences of unsustainable extractivism
Forest biological diversity	58	Species diversity, Genetic diversity	Role in ecosystem functionality and resilience
Tropical forests	42	Biodiversity hotspots	Challenges and conservation priorities
Subtropical forests	30	Ecosystem services, Climate regulation	Importance in global ecological balance
Boreal forests	28	Carbon sequestration, Biodiversity	Role in Climate Mitigation and biodiversity conservation
Natural Capital and extractivism	37	Economic valuation, Resource extraction	Impact on ecosystem services and sustainability
Deforestation and alteration of biodiversity	75	Habitat destruction, Species extinction	Long-term effects on global biodiversity
Interrelation and exchange of nutrients in networks	47	Nutrient Cycle, Ecosystem interdependence	Basis for forest productivity and health
Disorganized development versus organized development	25	Sustainable development, Land use planning	Effects on forest conservation and resource use
Nitrogen-fixing microorganisms	34	Nitrogen cycle, Soil fertility	Contribution to ecosystem nutrient dynamics
Impairment of cascade nutrient exchange	21	Pollution, Soil degradation	Impact on ecosystem nutrient cycles and productivity
Vadose zone and hydric sequences	16	Water cycle, Soil moisture	Influence on plant growth and ecosystem dynamics
Modification of water cycles	38	Climate change, Deforestation	Effects on hydrological systems and forest health
Cascading ecological effects	30	Ecosystem interconnections	Consequences of disruptions in ecological networks
Importance of public policies	56	Policy interventions, Conservation strategies	Role in regulating extractivism and protecting forests
Regulation of the extractivist framework	39	Legal frameworks, Governance	Approaches to sustainable resource management
Institutions and governance	48	Policy effectiveness, Institutional roles	Impact on environmental regulation and enforcement
Extractivism vs. Sustainability	33	Economic models, Environmental sustainability	Challenges in balancing resource use with conservation
Regulation of extractivism phenomena	22	Policy development, Environmental law	

**Table 2:** Synthesis of some characteristics of boreal, tropical, and subtropical forests.

Forest Region	Boreal Forests	Tropical Forests	Subtropical Forest
**Location**	Located in the north of the northern hemisphere. Russia (Siberia, European part); Canada (Yukon, British Columbia, to Newfoundland and Labrador); United States (Alaska); Sweden (North and center); Finland (North and East); Norway (North); Iceland Estonia; Latvia; Lithuania Kazakhstan (North); Mongolia (North).	Approximately 85 countries contain tropical forest ecosystems that cover 18 million km^2^. Brazil (Amazon rainforest); Indonesia (Sumatra, Borneo, New Guinea); Democratic Republic of the Congo (Congo Forest); Peru (Amazon rainforest); Colombia (Amazon Region); Venezuela (Amazon jungle, Orinoco Forests); Malaysia (Peninsular and island); Papua New Guinea; Bolivia (Amazon rainforest); Madagascar India (Northeast, western Ghats); Australia (Northern Queensland); Mexico (South, Yucatan Peninsula, Chiapas); Thailand	It is normally located between 23.5° and 35° latitude in both hemispheres. United States (Southeast, Florida, Georgia); China (South, Yunnan); Australia (East Coast, New South Wales, Queensland); India (Northeast, Western Ghats); Brazil (South, Paraná, São Paulo); Argentina (North, Misiones); Mexico (South, Chiapas, Veracruz); South Africa (East Coast, KwaZulu-Natal); Japan (South, Kyushu); New Zealand (North Island, Northland).
**Climate**	Extreme cold with temperatures that can drop to −45 degrees Celsius and short growing seasons.	Warm and humid with year-round growing seasons.	Generally warm with high humidity. Two different seasons: a hot and humid summer and a cooler and drier winter.
**Vegetation**	Forest soils are usually low in fertility and acidic, with a thin A horizon. These forests are dominated by conifers, spruce, pine, and larch, along with birch and poplar.	Various plant species and dense vegetation. Generally, tropical forest ecosystems extend further towards the poles, where humidity is adequate.	Diverse and dense, with a variety of trees, shrubs, and undergrowth. Evergreen broadleaf dominates these forests.
**Species Diversity**	Lower species diversity	Extremely high species diversity	The high diversity of species varies depending on the specific region.
**Dominant Fauna**	Moose, caribou, wolves, bears, owls.	Jaguars, monkeys, various birds, insects, and species of mushrooms.	Monkeys, tigers, snakes and numerous species of birds.
**Impact on Biodiversity**	Influenced by recurrent disturbances (fires, insect infestations). Habitat fragmentation causes species to be moved.	It houses the greatest biodiversity on the planet, which is why they are the true thermometer of the planet. This forest helps stabilize the world’s climate	These forests play a crucial role in the maintenance of biodiversity. They serve as vital refuges for migratory species. Help stabilize the global climate
**Carbon Storage**	Important for carbon storage	Carbon storage and oxygen production.	Important carbon reservoirs
**Importance of the Ecosystem**	One of the world’s leading providers of ecosystem services. Storage of carbon and fresh water.	High biodiversity, ecological services, and climate balance	It is important to maintain ecological and climatic balance.

## Data Availability

No new data were created.
